# Optical Beam Steerable Visible Light Communication (VLC) System Supporting Multiple Users Using RGB and Orthogonal Frequency Division Multiplexed (OFDM) Non-Orthogonal Multiple Access (NOMA)

**DOI:** 10.3390/s22228707

**Published:** 2022-11-11

**Authors:** Wahyu Hendra Gunawan, Chi-Wai Chow, Yang Liu, Yun-Han Chang, Chien-Hung Yeh

**Affiliations:** 1 Department of Photonics & Graduate Institute of Electro-Optical Engineering, College of Electrical and Computer Engineering, National Yang Ming Chiao Tung University, Hsinchu 30010, Taiwan; 2 Department of Photonics & Graduate Institute of Electro-Optical Engineering, College of Electrical and Computer Engineering, National Chiao Tung University, Hsinchu 30010, Taiwan; 3 Philips Electronics Ltd., N.T., Hong Kong; 4 Department of Photonics, Feng Chia University, Taichung 40724, Taiwan

**Keywords:** optical wireless communication (OWC), visible light communication (VLC), orthogonal frequency division multiplexing (OFDM), laser diode (LD)

## Abstract

In order to achieve high-capacity visible light communication (VLC), five dimensions in physics, including frequency, time, quadrature modulation, space, and polarization can be utilized. Orthogonality should be maintained in order to reduce the crosstalk among different dimensions. In this work, we illustrate a high-capacity 21.01 Gbit/s optical beam steerable VLC system with vibration mitigation based on orthogonal frequency division multiplexed (OFDM) non-orthogonal multiple access (NOMA) signals using red, green, and blue (RGB) laser-diodes (LDs). The OFDM-NOMA can increase the spectral efficiency of VLC signal by allowing high overlapping of different data channel spectra in the power domain to maximize the bandwidth utilization. In the NOMA scheme, different data channels are digitally multiplexed using different levels of power with superposition coding at the transmitter (Tx). Successive interference cancellation (SIC) is then utilized at the receiver (Rx) to retrieve different power multiplexed data channels. The total data rates (i.e., Data 1 and Data 2) achieved by the R/G/B OFDM-NOMA channels are 8.07, 6.62, and 6.32 Gbit/s, respectively, achieving an aggregated data rate of 21.01 Gbit/s. The corresponding average signal-to-noise ratios (SNRs) of Data 1 in the R, G, and B channels are 9.05, 9.18 and 8.94 dB, respectively, while that of Data 2 in the R, G, and B channels are 14.92, 14.29, and 13.80 dB, respectively.

## 1. Introduction

The radio-frequency (RF) bands have been nearly consumed, and using the optical frequency band could be a future-proof solution for the future of wireless communication systems [1]. Visible light communication (VLC), also known as optical wireless communication (OWC) using the visible light spectrum, can provide high-speed, high-privacy, high-flexibility, license-free, and electromagnetic interference (EMI) free wireless transmissions. VLC is also regarded as a promising candidate for the future 6G mobile networks [2]. It offers many unique transmission characteristics making it applicable in different scenarios, such as integrated lighting and communication [3,4], free-space optical communication (FSO) [5,6], underwater communication [7,8,9], optical camera communication [10,11], and visible light positioning (VLP) [12,13]. One main limitation of the VLC systems is the narrow modulation bandwidth of the optical light sources, such as the light emitting diodes (LEDs). Different schemes [14,15] were proposed to enhance the transmission capacity. Using spectral efficient orthogonal frequency division multiplexing (OFDM) has attracted many attentions in the VLC systems [16,17].

Recently, many high-data rate VLC systems using LED transmitter (Tx) were revealed. Hsu et al. demonstrated 1.1 Gbit/s MIMO VLC transmission using three white-light LEDs [15]. Cossu et al. exhibited 3.4 Gbit/s wavelength division multiplexed (WDM) VLC system using red, green, and blue (RGB) LEDs [18]. Then, Lu et al. showed 6.36 Gbit/s VLC transmission combining WDM, RGB, LEDs, MIMO, and polarization multiplexing [19]. Chi et al. revealed 3.375 Gbit/s VLC transmission using RGB LEDs and 8-level pulse amplitude modulation (PAM8) [20]. Moreover, Zhu et al. reported 10.72 Gbit/s VLC system using 5 different color LEDs: red, green, blue, yellow, cyan (RGBYC) [21]. To further promote the VLC transmission data rate, coherent light sources, such as visible laser diode (LD) can be utilized. Watson et al. exhibited 2.5 Gbit/s VLC system using a GaN blue LD and OOK modulation [22]. Chi et al. further increased the data rate to nine Gbit/s using GaN blue LD with OFDM [23]. Recently, Wu et al. reported an eye-safe white-light 8 Gbit/s VLC system using RGB LDs [24]. Besides, Lu et al. demonstrated 11.1 Gbit/s VLC transmission using a red VCSEL and OFDM [25]. Wei et al. illustrated 20.231 Gbit/s VLC transmission using RGB, LDs, and OFDM [26]. Besides, Gunawan et al. illustrated 28.4 Gbit/s VLC work combining RGB, LDs, OFDM, and color-shift-keying (CSK) [27]. Furthermore, Chun et al. showed 35 Gbit/s VLC system using 4 different color LDs: violet, blue, green, and red [28]. In the above reported systems, high capacity can be achieved by utilizing five dimensions in physics, including frequency, time, quadrature modulation, space, and polarization.

In this work, we propose and illustrate a high capacity 21.01 Gbit/s optical beam steerable VLC system with vibration mitigation based on OFDM non-orthogonal multiple access (NOMA) signals using RGB LDs. The OFDM-NOMA can increase the spectral efficiency of VLC signal by allowing high overlapping of different data channel spectra in the power domain to maximize the bandwidth utilization. Different NOMA VLC systems have been reported in the literatures [29,30,31,32]; however, they are based on direct line-of-sight (LOS) demonstrations [29,30] or using large Tx field-of-view (FOV) to cover different users [31,32]. Here, our main contribution is to demonstrate an optical beam steerable OFDM-NOMA VLC system with vibration mitigation. Besides, RGB wavelength multiplexing is also applied to increase the transmission capacity. Pre-forward-error-correction (pre-FEC) and bit-error-rate (BER = 3.8 × 10^−3^) thresholds are fulfilled in all the channels. The proposed VLC system has potential applications for indoor optical wireless communication (OWC), providing a high data rate and secure channel in machine-to-machine (M2M) or Internet-of-Thing (IOT) networks for B5G/6G. When comparing with the infrared (IR) based OWC system, the proposed VLC system employs the visible spectrum for communication. It offers advantages, such as providing lighting and communication simultaneously, as well as offering the potential application of visible light positioning (VLP). In addition, VLC system offers easier optical beam alignment and allows communication in underwater.

## 2. Algorithm and Experiment

**Figure 1:**
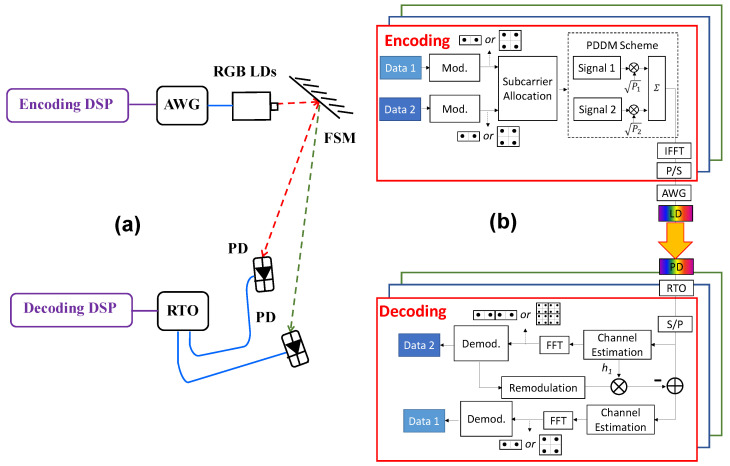
(**a**) Architecture of the RGB VLC systems using OFDM-NOMA. (**b**) Flow diagram and experimental setup of the proposed OFDM-NOMA VLC system using RGB LDs. IFFT: inverted fast-Fourier transform; P/S: parallel to serial; AWG: arbitrary waveform generator; S/P: serial to parallel. LD: laser diode; FSM: fast steering mirror; PD: photodiode; RTO: real time oscilloscope.

Figure 1a shows proposed architecture of the RGB VLC systems using OFDM-NOMA. A fast-steering mirror (FSM) can be used to steer the optical beam to different users or user-groups, achieving multiplexing. In the proposed scheme, RGB tri-color multiplexing, NOMA, and spatial multiplexing via FSM could be achieved. Figure 1b shows the flow diagram to reveal the encoding and decoding algorithms of the proposed OFDM-NOMA VLC system. Here, tri-color RGB LDs are used for the VLC transmission. For the OFDM-NOMA signal encoding at the Tx, two data channels (Data 1 and 2) are mapped into the different QAM formats. In the experiment, we used binary phase shift keying (BPSK) format and quadrature phase shift keying (QPSK) format for the analysis. The signals are allocated to different sub-carriers. Then, the two data channels at different levels of power are multiplexed using superposition code to produce OFDM-NOMA signal in Matlab^®^ program. Here, the constellation diagrams are shaped based on the power allocation to different data channels. This can be realized by multiplying each data signal by a specified power level *P*_1_ or *P*_2_. After this, OFDM encoding is performed, such as inverse fast Fourier transform (IFFT), parallel-to-serial (P/S) transposition, and cyclic prefix (CP) addition. The FFT size is 512, sub-carrier number is 125, and CP is 32. After the OFDM-NOMA signals are produced, they are applied to the RGB LDs via a digital-to-analog converter (DAC). In the experiment, an arbitrary waveform generator (AWG, Tektronix^®^ AWG 70001) acts as an ADC, which is used to transform the digital OFDM-NOMA signals into the real electrical waveforms to drive the RGB LDs. The AWG has an analog frequency range of 18 GHz and sampling rate of 50 GSample/s. The FSM (Optotune^®^ MR-15-30) used has the mirror diameter of 15 mm with silver coating, offering reflectivity >96% in wavelength window 400–2000 nm. The FSM achieves up to +/−25° mechanical tilt, which results in up to +/−50° optical deflection.

Here, the channel characteristics for the indoor VLC are analyzed. The response of the VLC channel with multiple-bounce power spectral distribution (PSD) [33] is described in Equation (1),

(1) $$h(t)=\sum_{k=0}^{\infty }h^{\left(k\right)}(t;\Phi_{n})$$

where $\Phi_{n}$ is different light source PSDs. The response after *k*-bounces is described in Equation (2),

(2) $$\begin{array}{l} h^{\left(k\right)}(t;\Phi_{n})=\int_{S}\left[L_{1}\ldots L_{k+1}\Gamma_{n}^{\left(k\right)}\mathrm{rect}(\frac{\theta_{k+1}}{FOV})\cdot \right.\\ \left.\delta (t-\frac{d_{1}+\ldots +d_{k+1}}{c})\right]dA_{ref} \end{array}$$

where

$$\begin{array}{l} L_{1}=\frac{A_{ref}(m+1)\cos^{m}\phi_{1}\cos\theta_{1}}{2\pi d_{1}^{2}},\cdots ,\\ L_{k+1}=\frac{A_{PD}\cos\phi_{k+1}\cos\theta_{k+1}}{\pi d_{k+1}^{2}}, \end{array}$$

is the path-losses. *c* is the speed of light, *S* is the reflector surface, and *A_ref_* is the reflecting element area. $\phi_{k}$ and $\theta_{k}$ are the angles of irradiance and incidence respectively. The received power is inversely proportional to the square of distance *d_k_*. $\Gamma_{n}^{\left(k\right)}$ is the reflected ray power after *k*-bounces. The PD receives optical power can be described in Equation (3) having an incidence angle smaller than the PD FOV.

(3) $$\mathrm{rect}(x)=\left\{\begin{array}{c} 1\;for\left|x\right|\leq 1,\\ 0\;for\left|x\right|>1. \end{array}\right.$$

In the LOS path, Equation (2) can be simplified as Equation (4),

(4) $$h^{\left(0\right)}(t;\Phi_{n})=L_{0}P_{n}\mathrm{rect}(\frac{\theta_{0}}{FOV})\delta (t-\frac{d_{0}}{c})$$

where

$$L_{0}=\frac{A_{PD}(m+1)\cos^{m}\phi_{0}\cos\theta_{0}}{2\pi d_{0}^{2}}$$

Without loss of generality, it is assumed there are two data signals, Data 1 and Data 2, with signals of *x_i_* (i.e., *i* = 1 and 2). *P_i_* (i.e., *i* = 1 and 2) is the power of the *ith* signal, and the total power is normalized as *P*_1_ + *P*_2_ = 1. The NOMA signal with the spectral overlaid signals having different power levels is illustrated in Equation (5).

(5) $$x=\sqrt{P_{1}}x_{1}+\sqrt{P_{2}}x_{2}$$

After the generation of the OFDM-NOMA signal in digital domain, it will be applied to a LD via the DAC (i.e., the AWG used in the experiment) to produce the optical OFDM-NOMA signal. The received optical signal can be expressed in Equation (6),

(6) y(t)=h(t)⊗x(t)+n(t)

where *x(t)* is the transmitted optical signal emitted the LD, *y(t)* is the received optical signal by the PD, *h(t)* is the impulse response of the channel obtained in Equation (1), and *n(t)* is the additive white Gaussian noise (AWGN) [34].

In the NOMA scheme, different data channels are digitally multiplexed using different levels of power with superposition coding at the Tx. Successive interference cancellation (SIC) [35,36,37] is then utilized at the receiver (Rx) to retrieve different power multiplexed data channels. As shown in Equation (5), different power domain multiplexed data channels should maintain a proper power ratio for the SIC process. The priority of the SIC process is based on the channel gain order. For instance, if Data 2 has a higher power than Data 1 (i.e., *P*_2_ > *P*_1_), the signal in Data 2, *x*_2_ is decoded first without the SIC process since the power of *x*_2_ is higher than *x*_1_. Then, the decoded *x*_2_ is used to obtain *x*_1_ in SIC process. The total channel capacity is also analyzed based on the Shannon-Hartley theorem. Equation (6) shows the total capacity *C,* which is equal to the sum of two digital power domain multiplexed data with capacities *C*_1_ and *C*_2_. *h*_1_ and *h*_2_ are the channel responses of Data 1 and Data 2, respectively. *B* and *P_N_* are bandwidth and noise power, respectively. In each wavelength channel, there is one set of Tx and Rx, and the Data 1 and Data 2 use the same wavelength channel; hence, bandwidth *B* and noise power *P_N_* are the same for the *C*_1_ and *C*_2_. As shown in Equation (7), the capacity *C*_2_ is affected by both the noise *P_N_* and the interference from the Data 1; while the capacity *C*_1_ is only affected by the noise *P_N__._*

(7) $$\begin{array}{l} C=C_{2}+C_{1}\\ =B\log_{2}\left(1+\frac{|h_{2}{|}^{2}P_{2}}{|h_{1}{|}^{2}P_{1}+P_{N}}\right)+B\log_{2}\left(1+\frac{|h_{1}{|}^{2}P_{1}}{P_{N}}\right) \end{array}$$

**Figure 2:**
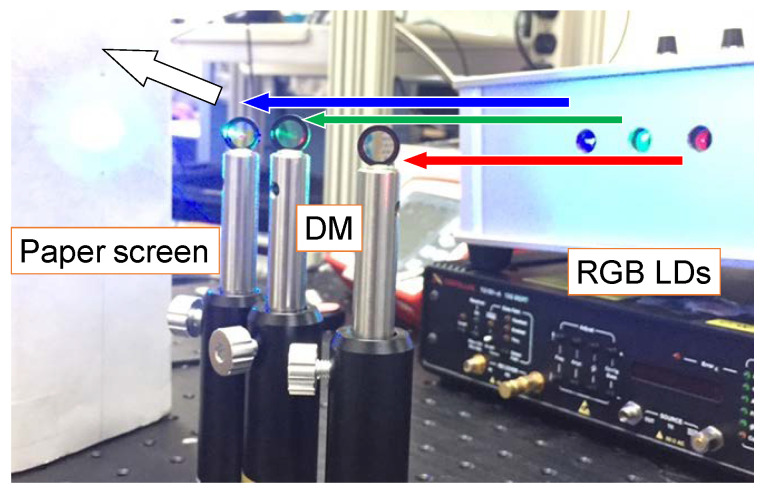
Experimental photo of the proposed OFDM-NOMA VLC system using RGB LDs. White light can be produced by combining the RGB light via a dichroic mirrors (DMs). Arrows indicate the directions of the R, G, B optical beams and the combined white light.

In this proof of concept experiment, there are 2 power domain multiplexed channels. The RGB channels have the typical wavelengths of 640, 514, 450 nm. The LDs are mounted in a home-made aluminum package with temperature control as illustrated in the photo in Figure 2. The three RGB channels emitted by the RGB LDs are combined via dichroic mirrors (DMs). In this proof-of-concept demonstration, the free-space transmission distance is about 2 m. As our RGB system is similar to the experiment work reported in [28], which has a coverage area of 39 m^2^ and a link distance of 4 m, we believe that our proposed VLC system could also support a transmission distance of 4 m. At the Rx side, color filters are used to separate the RGB color channels. After wavelength demultiplexing, each wavelength channel data is captured by a real time oscilloscope (RTO, LeCroy^®^ 816ZI-B) via a PIN PD (EOT^®^ ET-2030A), which has a 1.2 GHz 3-dB bandwidth. The PD has an active area diameter of 400 μm and full acceptance angle of 20°. The RTO acts as an ADC, which is used to transform the received electrical waveforms from the PD back to the digital domain for NOMA decoding. It has the analog bandwidth of 16 GHz and sampling rate of 80 GSample/s. The digital decoding is performed via LabVIEW^®^ and Matlab^®^ programs. As illustrated in Figure 1b, serial-to-parallel (S/P), zero-forcing mechanism, and channel estimation are needed to demultiplex the NOMA signals. Here, SIC process is utilized to demultiplex different NOMA data channels. The first step of the SIC process is to estimate the gain of the channel response, and then decode the strongest signal while considering all the other signals as noises. In this demonstration, Data 2 has the strongest level, and it will be decoded first while Data 1 is treated as noise. The second step is to re-modulate the estimated signal and multiply it by the channel response *h*_1_ before subtracting it from the total OFDM-NOMA signal. Then, the second strongest signal can be decoded. In this case, it is Data 1.

## 3. Results and Discussion

**Figure 3:**
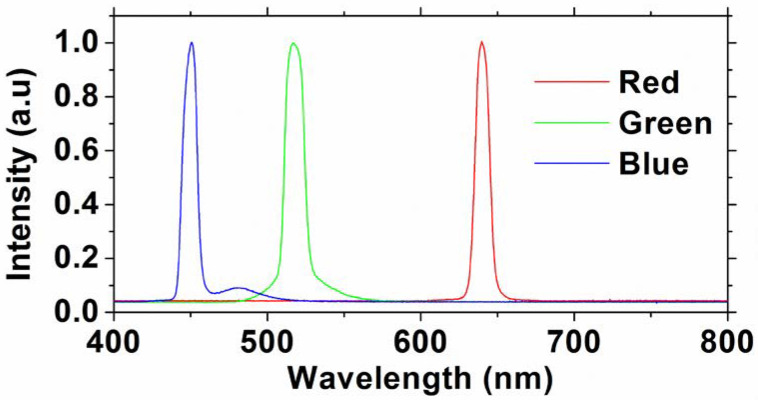
Experimental optical spectra of the RGB LDs.

**Figure 4:**
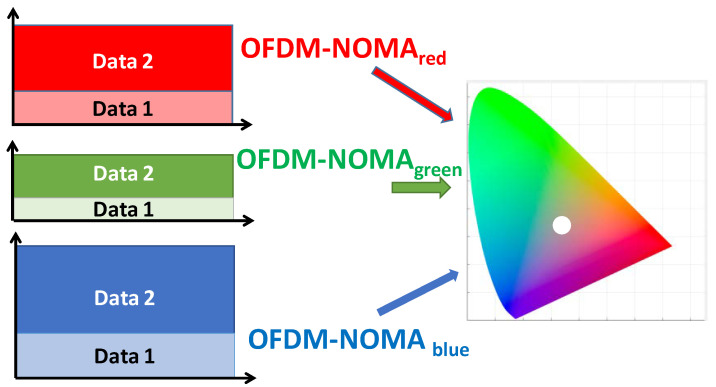
Concept of white light generation at the CIE 1931 chromaticity color gamut by the three color OFDM-NOMA channels.

**Figure 5:**
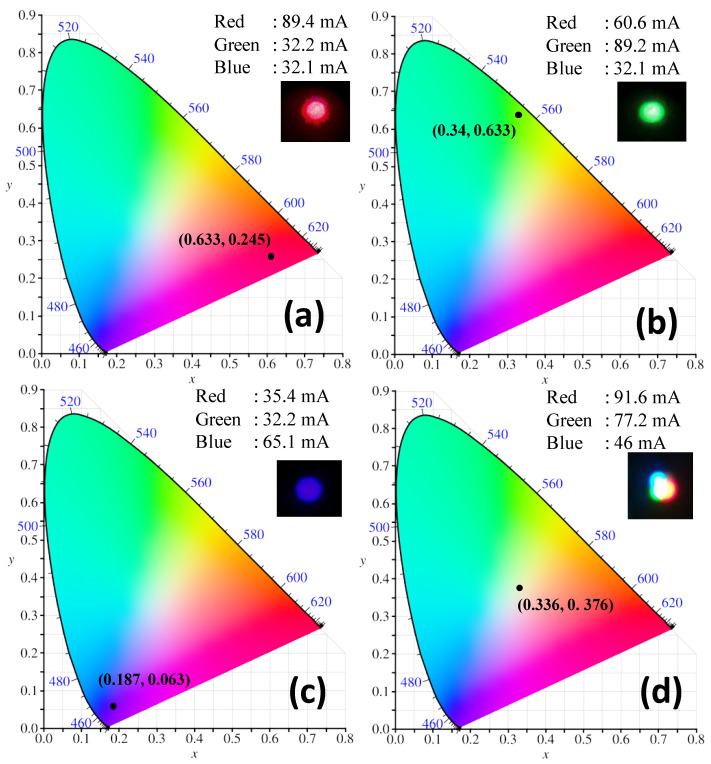
Experimentally measured CIE 1931 chromaticity color gamut at different driving currents for the RGB LDs respectively; showing colors of (**a**) red, (**b**) green, (**c**) blue and (**d**) white. Insets: photos of the color generated.

We study the color and the white-light generation of the proposed VLC systems. According to the CRC handbook [38], red, green, and blue colors have the wavelength ranges of 625–750 nm, 500–565 nm, 450–485 nm, respectively. The R, G, B wavelengths should be chosen within these wavelength ranges. Here, in the experiment, the LDs with wavelengths of 640 nm (red), 514 nm (green), and 450 nm (blue) are selected since they are commercially available and inexpensive. Figure 3 illustrates the experimental optical spectra of the RGB LDs with center wavelengths of 660, 514, and 450 nm, respectively. We can observe overlapping between the blue and green spectra. Figure 3 is measured directly at the RGB LD outputs, and at the Rx side, color filters with passband wavelengths of 450 nm, 520 nm, 640 nm, and bandwidths of 10 nm each are used to separate the RGB color channels. Hence, after the color filter, the crosstalk from adjacent color channels is negligible. Figure 4 illustrates the concept of color generation at the Commission internationale de l’éclairage (CIE) 1931 chromaticity color gamut by the three color channels [39]. By adjusting the relative optical powers among the OFDM-NOMA_red_, OFDM-NOMA_green_, and OFDM-NOMA_blue_ channels, different colors or white-light can be achieved. It is worth to mention that since the white light is produced by a relative optical power ratio among the OFDM-NOMA_red_, OFDM-NOMA_green_, and OFDM-NOMA_blue_ channels, but not by the absolute optical power in each color channel, if the signal-to-noise ratios (SNRs) for the three color channels are high enough, the Data 1 and Data 2 signals in each NOMA color channel can be decoded successfully. The SNR requirement will be studied later in this section. Besides, it is also worth mentioning that as the OFDM signal is modulated at much higher speed than the detectable flickering frequency of human eyes (i.e., ~ 100 Hz), no detectable color nor light fluctuation is observed in the experiment. Figure 5a–d show the experimentally measured CIE 1931 chromaticity color gamut at different driving currents for the RGB LDs, respectively. By adjusting the relative power ratio among the OFDM-NOMA_red_, OFDM-NOMA_green_, and OFDM-NOMA_blue_ channels, red, green, blue, and white color can be observed, as illustrated in the coordinates (*x*, *y*), the color gamut, and the inset photos.

**Figure 6:**
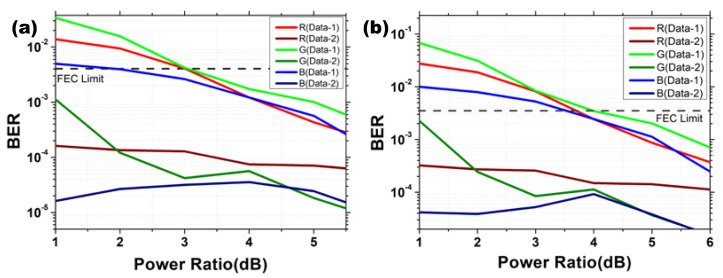
Experimental BER against different power ratios (Data 2: Data 1) of the OFDM-NOMA VLC systems using (**a**) BPSK and (**b**) QPSK.

As the two data channels are superimposed in power domain, their constellations are shaped based on the power allocation, a proper power ratio should be maintained in the OFDM-NOMA signal to satisfy the pre-FEC BER. In the experiment, we evaluate both the BPSK and QPSK formats. Figure 6a,b shows the experimental BERs against different power ratios of Data 2: Data 1 of the OFDM-NOMA VLC systems using BPSK and QPSK, respectively. It can be observed in both figures that the power ratio has a significant effect on Data 1, since it is the fundamental lower power channel. Changing the power ratio will greatly affect the shape of the constellation diagram producing high error rate during the SIC process. However, the power ratio has a small effect on the Data 2, since the constellation of the higher power Data 2 is shaped onto the fundamental constellations of Data 1. As shown in Figure 6a, when the power ratio of Data 2 to Data 1 in BPSK format is 4:1, the two power multiplexed data in all the RGB channels can satisfy the pre-FEC threshold. The BERs of Data 1 and Data 2 are: 1.21 × 10^−3^ and 7.46 × 10^−5^; 1.74 × 10^−3^ and 5.63 × 10^−5^; 1.22 × 10^−3^ and 4.60 × 10^−5^ for the R, G, B channels, respectively. As shown in Figure 6b, when the power ratio of Data 2 to Data 1 in QPSK format is 4:1, the two power multiplexed data in all the RGB channels can also satisfy the pre-FEC threshold. The BERs of Data 1 and Data 2 are 2.43 × 10^−3^ and 1.49 × 10^−4^; 3.47 × 10^−3^ and 1.13 × 10^−4^; 2.44 × 10^−3^ and 9.20 × 10^−5^ for the R, G, B channels, respectively.

**Figure 7:**
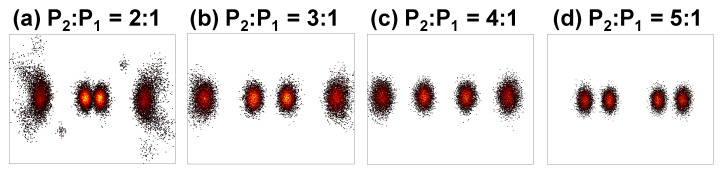
Experimental constellation diagrams of the power domain combined Data 1 and Data 2 OFDM-NOMA signals at different power ratios using BPSK.

**Figure 8:**
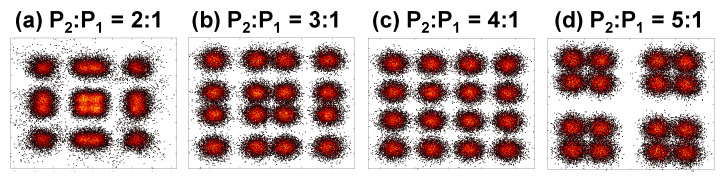
Experimental constellation diagrams of the power domain combined Data 1 and Data 2 OFDM-NOMA signals at different power ratios using QPSK.

Figure 7 and Figure 8 illustrate the experimental constellation diagrams of the power domain combined Data 1 and Data 2 OFDM-NOMA signals at different power ratios using BPSK and QPSK formats respectively. As discussed before, the constellations are shaped depending on the different power allocations; hence the Data 1 and Date 2 combined NOMA BPSK signal appears as 8-QAM while the Data 1 and Date 2 combined NOMA QPSK signal appears 16-QAM. The constellation diagrams in Figure 7 and Figure 8 also illustrate that when the power ratio of P_2_ to P_1_ is 4:1, the clear and well distinguished constellations can be observed. This is agree with the BER measurements shown in Figure 6a,b.

**Figure 9:**
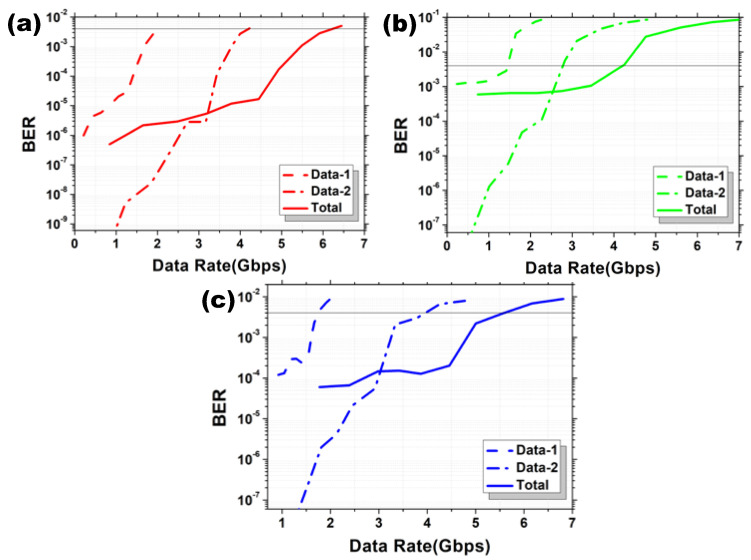
Experimental BER curves of the Data 1, Data 2, and the combined OFDM-NOMA signals for the (**a**) R, (**b**) G, and (**c**) B channels in BPSK format.

**Figure 10:**
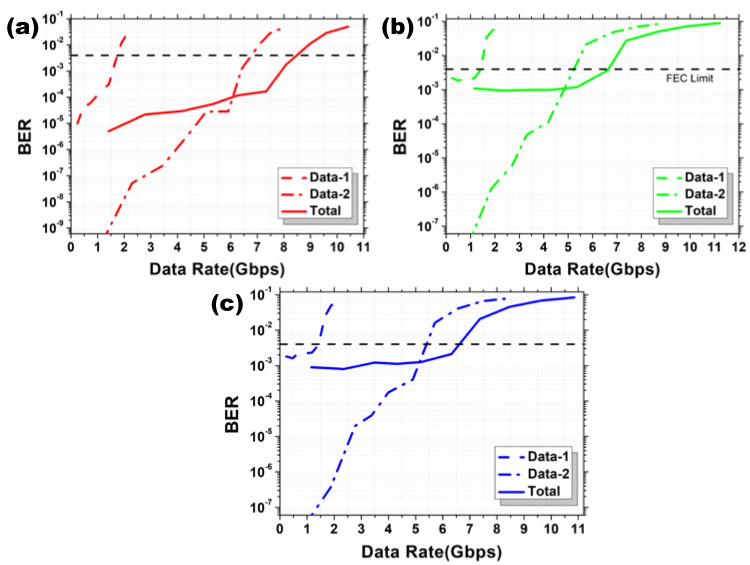
Experimental BER curves of the Data 1, Data 2, and the combined OFDM-NOMA signals for the (**a**) R, (**b**) G, and (**c**) B channels in QPSK format.

We study the maximum achievable data rates against BER of the OFDM-NOMA multiplexed Data 1 and Data 2 in different RGB channels, respectively. Figure 9a–c shows the experimental BER curves of the Data 1, Data 2, and the combined OFDM-NOMA signals for the RGB channels in BPSK format, respectively. We select the highest data rate signal fulfilling the pre-FEC BER threshold in each case. 5.92 Gbit/s at the BER 2.82 × 10^−3^ is obtained in the R channel, 4.25 Gbit/s at the BER 4.30 × 10^−3^ is obtained in the G channel, and 5 Gbit/s at the BER 2.2 × 10^−3^ is obtained in the B channel. Hence, a total data rate for the OFDM-NOMA RGB signal in BPSK is 15.17 Gbit/s. Figure 10a–c shows the experimental BER curves of the Data 1, Data 2 and the combined OFDM-NOMA signals for the RGB channels in QPSK format respectively. 8.07 Gbit/s at the BER 1.7 × 10^−3^ is obtained in the R channel, 6.62 Gbit/s at the BER 3.72 × 10^−3^ is obtained in the G channel, and 6.32 Gbit/s at the BER 2.1 × 10^−3^ is obtained in the B channel. Hence, a total data rate for the OFDM-NOMA RGB signal in QPSK is 21.01 Gbit/s.

The main limiting parameter of the speed of the VLC system is the direct modulation bandwidths of the R, G, and B LDs. The direct modulation bandwidth of the LD can be increased by using higher electrical driving current. However, the applied electrical direct-current (DC) bias should not be too high since the LD should be operated in the nonlinear region. Besides, as the OFDM-NOMA data has a high peak-to-average power ratio (PAPR), the applied electrical driving data should not be too high to avoid signal clipping. In the experiment, we have optimized the applied DC bias as well as the electrical driving data of the OFDM-NOMA for each LD. The total capacity of the VLC system can be increased by using polarization division multiplexing. As illustrated in [40] by using s-polarization and p-polarization of each color channel to carry different data, the total transmission capacity can be nearly doubled. Besides, adding more color channels via wavelength division multiplexing can also increase the total transmission capacity. For example, in [41], besides using RGB, the yellow (Y) color channel was included to produce a RGBY VLC system. Moreover, in [42], the violet (V) color channel was included to produce a RGBV VLC system.

**Figure 11:**
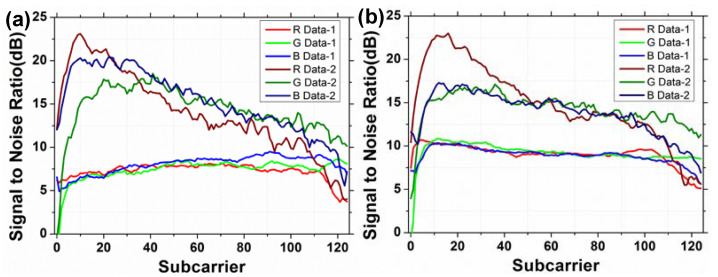
Experimental SNRs of the Data 1 and Data 2 over all the 125 OFDM subcarrier for R, G, and B channels in the OFDM-NOMA signals using (**a**) BPSK and (**b**) QPSK.

**Figure 12:**
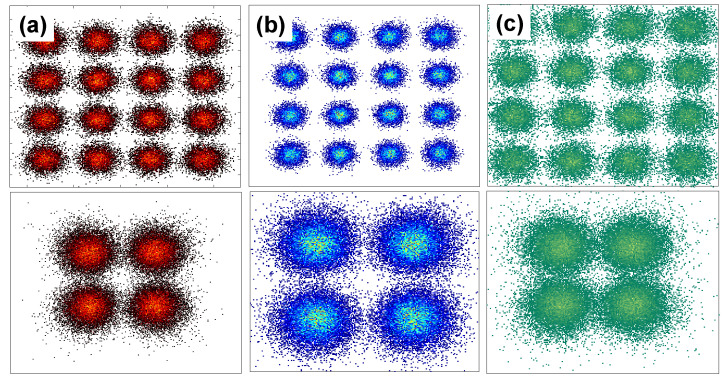
Experimental constellation diagrams for Data 1 and Data 2 for OFDM-NOMA (**a**) R, (**b**) G, (**c**) B channels.

Figure 11a,b illustrate the experimental SNRs of the Data 1 and Data 2 over all the 125 OFDM subcarrier for R, G, and B channels in the OFDM-NOMA signals using BPSK and QPSK formats, respectively. As shown in both figures, the SNRs for the Data 2 are higher than the Data 1 due to the higher power ratio. For the BPSK format shown in Figure 11a, the average SNRs of Data 1 in the R, G, B channels are 7.22 dB, 7.36 dB, and 8.01 dB, respectively. The average SNRs of Data 2 in the R, G, B channels are 14.09 dB, 14.36 dB, and 15.32 dB, respectively. For the QPSK format shown in Figure 11b, the average SNRs of Data 1 in the R, G, B channels are 9.05 dB, 9.18 dB, and 8.94 dB, respectively. The average SNRs of Data 2 in the R, G, B channels are 14.92 dB, 14.29 dB, and 13.80 dB, respectively. Besides, the corresponding constellation diagrams for Data 1 and Data 2 for OFDM-NOMA RGB channels in QPSK format are illustrated in Figure 12a–c, respectively. We can observe that the constellation diagrams are clear and well separated.

**Figure 13:**
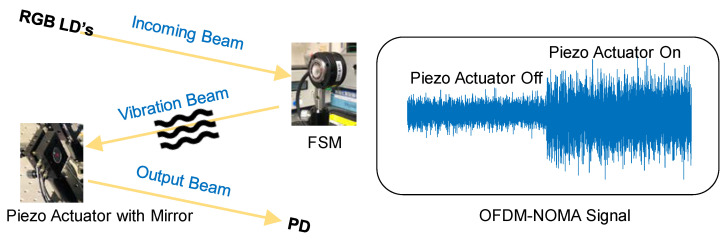
Illustration of vibration mitigation scheme for the OFDM-NOMA VLC system.

We apply vibration mitigation for the OFDM-NOMA VLC system as illustrated in Figure 13. Here, the FSM is used to emulate the vibration between the Tx and Rx via modulating the FSM at different frequencies. At the Rx side, a piezo actuator with mirror, a quadrature PD (QPD) and a feedback control are used to compensate the optical beam fluctuation via controlling the piezo actuator. The piezo mirror mount (Thorlabs^®^ KC1-PZ/M) has both piezo actuator control and manual mechanical control. The piezo angular travel range is +/−73 µrad with minimum step size of 0.3 µrad, while the manual mechanical angular range is +/−5°. The mirror (Thorlabs^®^ PF10-03-P01) mounted has a diameter of 25.4 mm. It has a silver coating with fused silica substrate, with >97.5% reflectance in wavelength window 450–2000 nm. We can observe in Figure 13 that when the piezo actuator is switched on, a much higher Rx optical power can be obtained. Table 1 summarizes the performance of the vibration mitigation scheme for the OFDM-NOMA VLC system. We can observe that when the driving frequencies are 1–2 Hz, the tilting amplitude of the FSM is large, and the optical beam cannot be tracked when the piezo actuator is ON. When the FSM driving frequencies are 3–5 Hz, the tilting amplitude of the FSM is relatively smaller, and the optical beam can be tracked within about 7.9 s. When the FSM driving frequencies >6 Hz, the FSM tiling as it is limited by the intrinsic response of the FSM. Hence, high power of OFDM-NOMA VLC signal can be received without the need of compensation.

## 4. Conclusions

In order to achieve high-capacity transmission, five dimensions in physics, including frequency, time, quadrature modulation, space, and polarization can be utilized. Orthogonality should be maintained in order to reduce the crosstalk among different dimensions. In this work, we illustrated a high capacity 21.01 Gbit/s optical beam steerable RGB VLC system using OFDM-NOMA signals, meeting the pre-FEC BER requirement. The OFDM-NOMA signal can increase the aggregated transmission capacity by allowing high overlapping of different data channel spectra in the power domain to maximize the bandwidth utilization. The data rates achieved by the OFDM-NOMA RGB channels in BPSK format were 5.92, 4.25, and 5 Gbit/s. Hence an aggregated data rate of 15.17 Gbit/s was achieved. On the other hand, the data rates achieved by the OFDM-NOMA RGB channels in QPSK format were 8.07, 6.62, and 6.32 Gbit/s, respectively. Hence, an aggregated data rate of 21.01 Gbit/s was achieved. In addition, for the QPSK format, the average SNRs of Data 1 in the R, G, and B channels were 9.05, 9.18, and 8.94 dB, respectively, while that of Data 2 in the R, G, and B channels are 14.92, 14.29, and 13.80 dB. Here, we also studied the color generated by adjusting the relative power ratio among the RGB channels.

## Figures and Tables

**Table 1:** Performance of vibration mitigation scheme.

FSM Freq. (Hz)	Rx Amp. (mV)	Tracked by Piezo	Time for Tracking and Feedback (s)
1	32	No	-
2	44	No	-
3	207	Yes	8
4	180	Yes	7.8
5	177	Yes	7.9
6–10	180	No need	No need

## Data Availability

The data presented in this study are available from the first author upon request.
